# Analyzing Google COVID-19 Vaccine Intent Search Trends and Vaccine Readiness in the United States: Panel Data Study

**DOI:** 10.2196/55422

**Published:** 2024-07-29

**Authors:** Kenneth W Moffett, Michael C Marshall, Jae-Eun C Kim, Heather Dahlen, Benjamin Denison, Elissa C Kranzler, Morgan Meaney, Blake Hoffman, Ivica Pavisic, Leah Hoffman

**Affiliations:** 1 Fors Marsh Arlington, VA United States

**Keywords:** information-seeking behavior, COVID-19, internet use, vaccination, vaccine hesitancy

## Abstract

**Background:**

Factors such as anxiety, worry, and perceptions of insufficient knowledge about a topic motivate individuals to seek web-based health information to guide their health-related decision-making. These factors converged during the COVID-19 pandemic and were linked to COVID-19 vaccination decision-making. While research shows that web-based search relevant to COVID-19 was associated with subsequent vaccine uptake, less is known about COVID-19 vaccine intent search (which assesses vaccine availability, accessibility, and eligibility) as a signal of vaccine readiness.

**Objective:**

To increase knowledge about vaccine intent search as a signal of vaccine readiness, we investigated the relationship between COVID-19 vaccine readiness and COVID-19 vaccine intent relative search volume on Google.

**Methods:**

We compiled panel data from several data sources in all US counties between January 2021 and April 2023, a time during which those with primary COVID-19 vaccinations increased from <57,000 to >230 million adults. We estimated a random effects generalized least squares regression model with time-fixed effects to assess the relationship between county-level COVID-19 vaccine readiness and COVID-19 vaccine intent relative search volume. We controlled for health care capacity, per capita COVID-19 cases and vaccination doses administered, and sociodemographic indicators.

**Results:**

The county-level proportions of unvaccinated adults who reported that they would wait and see before getting a COVID-19 vaccine were positively associated with COVID-19 vaccine intent relative search volume (β=9.123; *Z*=3.59; *P*<.001). The county-level proportions of vaccine-enthusiast adults, adults who indicated they were either already vaccinated with a primary COVID-19 vaccine series or planned to complete the vaccine series soon, were negatively associated with COVID-19 vaccine intent relative search volume (β=–10.232; *Z*=–7.94; *P*<.001). However, vaccine intent search was higher in counties with high proportions of people who decided to wait and see and lower in counties with high proportions of vaccine enthusiasts.

**Conclusions:**

During this period of steep increase in COVID-19 vaccination, web-based search may have signaled differences in county-level COVID-19 vaccine readiness. More vaccine intent searches occurred in high wait-and-see counties, whereas fewer vaccine intent searches occurred in high vaccine-enthusiast counties. Considering previous research that identified a relationship between vaccine intent search and subsequent vaccine uptake, these findings suggest that vaccine intent search aligned with people’s transition from the wait-and-see stage to the vaccine-enthusiast stage. The findings also suggest that web-based search trends may signal localized changes in information seeking and decision-making antecedent to vaccine uptake. Changes in web-based search trends illuminate opportunities for governments and other organizations to strategically allocate resources to increase vaccine uptake. Resource use is part of the larger public policy decisions that influence vaccine uptake, such as efforts to educate the public during evolving public health crises, including future pandemics.

## Introduction

### Background

Individuals regularly seek health information to guide their health-related decision-making about varied topics, including cancer screening [1], electronic cigarette use [2], vaccination against human papillomavirus [3], and COVID-19 [4]. People acquire this information from many sources, including their medical providers [5]; traditional media sources such as broadcast television and radio [6]; and digital media sources such as apps, websites, and streaming video and audio [7]. Many people search for web-based health-related information [7,8] using search engines such as Google (Google LLC), which has >90% of the web-based search market share in the United States [9,10]. Individuals are more likely to seek knowledge on the web when they perceive online information to be available, high quality, trustworthy, useful, and credible [11,12].

People search for information when they have insufficient knowledge about a topic or perceive a knowledge gap [13]. In addition, worry and anxiety are associated with higher levels of health information seeking [14,15]. These conditions can lead to hesitancy in making health decisions because people perceive limitations in their knowledge, hampering their ability to successfully mitigate risks [16-18]. Health information seeking–related worry, anxiety, and perceived limitations in knowledge converged when the COVID-19 pandemic began in 2020, as the public initially knew little about SARS-CoV-2 (the virus that causes COVID-19) [19,20]. High levels of anxiety and worry about COVID-19 extended to COVID-19 vaccination decision-making [13], with a substantial proportion of the public initially hesitant about whether (or when) to get a COVID-19 vaccine [21,22]. High levels of COVID-19 anxiety and increases in depressive symptoms and stress were also present among health care workers [23].

In response to COVID-19 and COVID-19–related mortality, countries used several public policy responses during the pandemic emergency period, including increasing health care spending [24], strengthening early warning systems and adding robust contact tracing systems [25], and supporting research to develop COVID-19 vaccines and treat COVID-19 [25]. Once COVID-19 vaccines were created and approved, many high-income countries could vaccinate their populations more quickly [26], which reduced COVID-19 mortality [27]. Conversely, countries with fewer resources and lower access to COVID-19 vaccines could not vaccinate their populations as quickly [28].

As countries implemented public policy responses during the early stages of the COVID-19 pandemic, researchers observed surges in web-based COVID-19–related content [29,30] and an increase in COVID-19–related searches [31]. Using data from Google Trends (Google LLC), which quantifies relative interest in a search topic, researchers identified spikes in the number of new COVID-19 cases that coincided with increases in relative COVID-19 search activity [4,32]. COVID-19-related searches included general searches about COVID-19 as well as specific search queries, such as those about the safety and efficacy of COVID-19 vaccines, unfounded concerns regarding ethylmercury content in vaccine preservatives (there are no preservatives in COVID-19 vaccines [33]), and unfounded links [34] between vaccination and autism (this claim has been proven false [4,35]). Moreover, from March to June 2020 (in the early stages of the pandemic), high levels of anxiety and depression in the population were associated with increases in COVID-19 vaccine searches in the United States [20].

COVID-19–related searches also increased with the announcement and publication of scientific advancements in COVID-19 vaccine development [4,36]. One study used a machine learning methodology to show that people who were clustered into a group that was more likely to gather information on the web from multiple sources had longer life expectancies, were college educated, had higher per capita incomes, lived in metropolitan areas, and were less likely to be vaccine hesitant [37], suggesting that web-based searches related to COVID-19 may have influenced individuals’ COVID-19 vaccination decisions. Studies using Google Trends data found a positive association between the amount of peer-reviewed scientific research about Pfizer and Moderna COVID-19 vaccines and information-seeking searches about these vaccines [36] and an association between the December 2020 US emergency use authorization and an immediate increase in Google search volume about the unfounded link [38] between side effects of the vaccine and fertility [39,40].

Moreover, insights gathered from Google COVID-19 vaccination search index trend data demonstrated that increased interest in this topic was associated with the number of new COVID-19 vaccinations administered over the subsequent 3 weeks and with vaccination rates observed months later [41]. The Google COVID-19 vaccination search index trend data were processed in a more specified manner than data available from Google Trends to make them more usable for researchers and practitioners. We used Google COVID-19 vaccination search index trend data in this analysis and not data from Google Trends.

**Figure 1:**
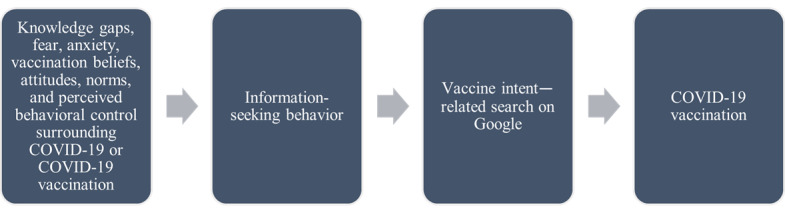
Information-seeking process for COVID-19 vaccination.

To better understand the relationship between COVID-19-related web-based search and COVID-19 vaccination intentions and uptake, we investigated the potential association between COVID-19 vaccine readiness and the volume of COVID-19 vaccine intent–related searches on Google between January 2021 and April 2023. During this time frame, the number of adults with primary COVID-19 vaccinations increased from <57,000 to >230 million [42]. Vaccine readiness is a composite measure of vaccine intention and behavior. Vaccine intent–related searches included those on the accessibility, availability, and eligibility of for COVID-19 vaccines and those on topics such as “COVID-19 vaccine near me” [43]. Vaccine intent–related searches represent what could be the first step after making a decision to get vaccinated (Figure 1), as individuals seek information on how to get their COVID-19 vaccination. Evidence indicates that increased vaccine intent searches preceded higher COVID-19 vaccination rates [41].

Researchers have developed theories and models to explain health behavior uptake that have empirical support to elaborate the conditions under which people are more likely to seek information. The theory of planned behavior (TPB) posits that the decision to engage in a volitional behavior is primarily influenced by behavioral intention, which is shaped by a combination of factors such as attitudes, social norms, and perceived behavioral control [44]. The Planned Risk Information Seeking Model (PRISM) builds upon the TPB by focusing on individuals who perceive limitations in their knowledge that can help them mitigate risks [16-18]. More specifically, PRISM suggests that people who perceive limitations in their knowledge are more likely to seek information to increase their knowledge and address perceived knowledge insufficiency [16].

Altogether, these theories suggest that people move through an information-seeking process, as outlined in Figure 1. This figure illustrates the process by which individuals moved from deciding to seek additional information about COVID-19 or COVID-19 vaccination to getting a COVID-19 vaccine.

In the first stage, multiple factors drove individuals’ information-seeking behavior to address COVID-19 vaccination knowledge gaps, including fear and anxiety about COVID-19 or COVID-19 vaccination [13]; vaccination beliefs [45]; and, more broadly, their relevant attitudes, norms, and perceived behavioral control [16]. As vaccine-hesitant individuals gained more information through web-based searches to fill perceived or actual knowledge gaps and moved from being vaccine hesitant to vaccine ready, these theories suggest that individuals were more likely to perform vaccine intent–related searches as the final step of a decision-making and information-seeking process that culminated in getting a COVID-19 vaccine to mitigate the risk of COVID-19–related harm to their health.

### Objectives

This study investigated the following research question: Is there an association between COVID-19 vaccine readiness and the volume of COVID-19 vaccine intent–related searches on Google?

Google searches related to vaccine intent excluded searches for COVID-19 vaccine safety and general searches about the COVID-19 vaccine [46]. We examined county-level proportions of 3 groups of adults based on vaccine readiness: vaccine enthusiasts, those who wanted to wait and see, and those who had no intention to get vaccinated.

We expected that vaccine enthusiasts, people who had already gotten their primary COVID-19 vaccination or intended to get vaccinated soon, would be less likely to search for COVID-19 vaccine intent information on the web because they had already made or enacted an affirmative COVID-19 vaccination decision for themselves. Accordingly, we hypothesized a negative relationship between the county-level proportion of adults who were vaccine enthusiasts and the county-level relative COVID-19 vaccine intent search volume on Google (hypothesis 1).

Conversely, we expected that individuals in the wait-and-see group initially lacked the information they needed to make a vaccination decision and, therefore, would be more likely to search for COVID-19 vaccine information on the web to facilitate decision-making. As these individuals became vaccine ready, we expected that they would be more likely to perform COVID-19 vaccine intent searches as a final step in a decision-making and information-seeking process. That process concluded when they got a COVID-19 vaccine. Accordingly, we hypothesized a positive relationship between the county-level proportion of adults who were in the wait-and-see group and the county-level relative COVID-19 vaccine intent search volume on Google (hypothesis 2).

## Methods

### Ethical Considerations

Institutional review board approval for this research was not required because it did not meet the US Department of Health and Human Services’ (HHS) definition of human subjects research. All data for this study came from deidentified, publicly available sources.

### Data

Data were drawn from several publicly available sources, including Google vaccine search insights data [43], the HHS Monthly Outcome Survey (MOS) [47], the US Centers for Disease Control and Prevention’s (CDC’s) Weekly United States COVID-19 Cases and Deaths by County database [48], the HHS Area Health Resources Files (AHRFs) [49], the US Federal Communications Commission’s (FCC’s) Form 477 County Data on Internet Access Services [50], and the Subnational Ideology and Presidential Election Estimates data set [51,52].

COVID-19 vaccine intent search data were derived from Google’s weekly vaccine search insights data by county. These data provided insights on topics related to COVID-19 vaccine availability, accessibility, and eligibility [43]. Hereafter, we refer to these searches as the COVID-19 vaccine intent relative search volume. The MOS provided data on the proportions of the population who were in each vaccine readiness group: vaccine enthusiasts, wait and see, and no intent to get vaccinated [47]. These groups are more defined in detail in the *Measures of Variables* section. The CDC data contained the number of COVID-19 cases per capita and the number of COVID-19 vaccination doses per capita [48]. The HHS AHRFs contained county-level public health infrastructure and demographic data [49]. The publicly available FCC data reflected the rates of internet access in each US county [50]. Finally, data from the American Ideology Project provided county-level political context measures (for 2016 and 2020) [51,52].

We used these data to investigate the link between COVID-19 vaccine readiness and COVID-19 vaccine intent relative search volume. The data were recorded or aggregated for each month and each county, so the unit of analysis was the county-month.

### Measures of Variables

The dependent variable was the monthly county-level COVID-19 vaccine intent relative search volume from January 4, 2021, to April 24, 2023, in the United States. Google weekly vaccine search insights data provided weekly vaccination intent–related searches conducted by individuals at the zip code level. These searches included those related to the availability, accessibility, and eligibility of COVID-19 vaccines [43]. These data were normalized with a minimum value of 0 to indicate no relative interest. We aggregated these data using the median relative intent search index indicator by county and month. We used median values instead of mean values because they were less subject to being skewed by outliers. More information about this measure is provided in the *Data Sources and Analytic Method* section of Multimedia Appendix 1 [53-58] and on Google’s COVID-19 Vaccination Search Insights page [43,59].

Our independent variables were 2 monthly, county-level measures of the proportions of people in each of the 2 COVID-19 vaccine readiness categories: vaccine enthusiasts and wait and see. Vaccine enthusiasts indicated that they were “already vaccinated or reported that they will get a vaccine as soon as they can” [47]. The wait-and-see group [47] reported “that they will wait to get a primary series vaccination for one or more reasons” [47]. Our reference category (the no-intent-to-get-vaccinated group) was the proportion of people who were unvaccinated and reported that they would never get a COVID-19 vaccine [47]. These measures were constructed using small area estimates that were based on covariates in the MOS. More information on the construction of these measures and the underlying MOS data sets is provided in the *Data Sources and Analytic Method* section of Multimedia Appendix 1 and on the HHS health database [47].

Internet access is not distributed equally across the United States because this distribution is largely driven by differences in affordability and density [60]. Although rural areas have less access and lower broadband wired speeds than urban areas, more people without broadband access live in urban areas than in rural areas [60-62]. To consider the potential influence of county-level variation in internet access on COVID-19 vaccine intent relative search volume, we aggregated fixed and broadband internet coverage rates by county using the most recent FCC data from 2021 [50] to calculate mean internet access.

A community’s capacity to provide health care services to its constituents could influence an individual’s perceived access to COVID-19 treatment and thereby influence their intention to get (or the uptake of) a COVID-19 vaccine [63,64]. To account for the potential influence of county-level health care capacity on COVID-19 vaccine readiness, our analyses controlled for 3 measures relevant to health care capacity in each county, as provided by the HHS AHRFs: the number of public health research facilities per county; the number of staffed intensive care unit hospital beds per 100,000 people; and the number of primary care physicians per county square mile [49]. Public health research facilities included the number of public health research centers, facilities, hospitals, universities, and similar institutions [49]. Through their research and outreach activities in their local area, public health research centers provide offline health information, may facilitate an environment that encourages access to vaccination, or may even have qualified staff who devote time to delivering COVID-19 vaccines. Primary care physicians included family medicine, general practitioners, and internists [49]. We expected that people would have better access to offline health information and perceive better access to COVID-19 vaccines as the numbers of public health research centers, intensive care unit beds, and primary care physicians per county increase.

To account for the potential influence of COVID-19 cases and vaccinations on COVID-19 vaccine intent relative search volume, we gathered weekly county-level CDC COVID-19 case and vaccination administration data [48]. We then normalized aggregated numbers of cases and vaccinations by computing per capita (100,000 people) measures. We calculated the differences in each of these quantities between the current month and the previous month and lagged each difference by 1 month, with the expectation that changes in COVID-19 cases and vaccinations in the previous month could be associated with subsequent changes in COVID-19 vaccine intent relative search volume. More specifically, a higher number of COVID-19 cases likely increased personal risk perception for getting COVID-19 and motivated information seeking about COVID-19 prevention behavior such as vaccinations. A higher number of COVID-19 vaccinations was likely to positively influence perceived social norms of getting vaccinated, which, in turn, would have affected vaccination information seeking.

Previous research has shown that attitudes and beliefs about COVID-19 vaccination and COVID-19 vaccine uptake differ based on sociodemographic characteristics, including race or ethnicity, income, and political ideology [21,22,37]. To account for the potential influence of sociodemographic characteristics on COVID-19 vaccine readiness, we gathered data from the HHS AHRFs on the county-level proportions of people who reported that they identified with the following racial or ethnic groups in the 2020 Census: non-Hispanic Black, Hispanic, non-Hispanic Asian and Pacific Islander, and non-Hispanic American Indian and Alaska Native. We also gathered HHS AHRF data on the per capita income of each county in 2021.

The models included 2 indicators that captured political context, developed from the American Ideology Project [51,52]. First, we created a measure for being an electoral pivot county, in which we examined the change in the US presidential election results between the 2016 and 2020 elections. The electoral pivot measure was a dichotomous variable coded as 1 for counties that contained a plurality of the population who voted for the Republican presidential candidate in the 2016 general election but voted for the Democratic presidential candidate in the 2020 general election and coded as 0 for those counties that did not meet this condition. Second, we included a measure that captured the change in county-level political ideology from 2016 to 2020. This measure was scaled such that higher values indicated more conservative counties in 2020 compared to 2016. More details about both measures are provided in the *Data Sources and Analytic Method* section of Multimedia Appendix 1.

### Models and Data Analysis Procedure

To assess the relationship between COVID-19 vaccine readiness and COVID-19 vaccine intent relative search volume, we estimated a random effects generalized least squares regression model with time-fixed effects and clustered robust SEs by county. This methodology allowed us to model longitudinal data in which the dependent variable, county-level COVID-19 vaccine intent relative search volume, has a lower bound (0) but no theoretical upper bound. Using fixed effects allowed us to disentangle any relationships between independent and dependent variables while controlling for the effects of time. The SEs were clustered to adjust for uneven variance in COVID-19 vaccine intent relative search volume across counties [65]. We used the natural logs of mean internet access, public health research facilities, access to primary care physicians, monthly change in COVID-19 cases per capita, monthly change in COVID-19 vaccination doses per capita, and income per capita because they were not normally distributed in their raw forms.

After estimating the statistical model, we performed predictive margins tests to estimate the substantive impact of both COVID-19 vaccine readiness variables (monthly change in vaccine enthusiasts and monthly change in the wait-and-see group) more precisely on the COVID-19 vaccine intent relative search volume. More details about our analytic method and the predictive margins tests are available in the *Data Sources and Analytic Method* section of Multimedia Appendix 1.

We used a stepwise approach to arrive at the model, which provided an internal consistency check on the primary expectations with respect to control variable inclusion or exclusion. More details about how we implemented this approach and arrived at the model that we reported are available in the *Stepwise Regression Results* section of Multimedia Appendix 1. We conducted the analyses using Stata (version 17; StataCorp) [66].

## Results

### Descriptive Analyses

**Figure 2:**
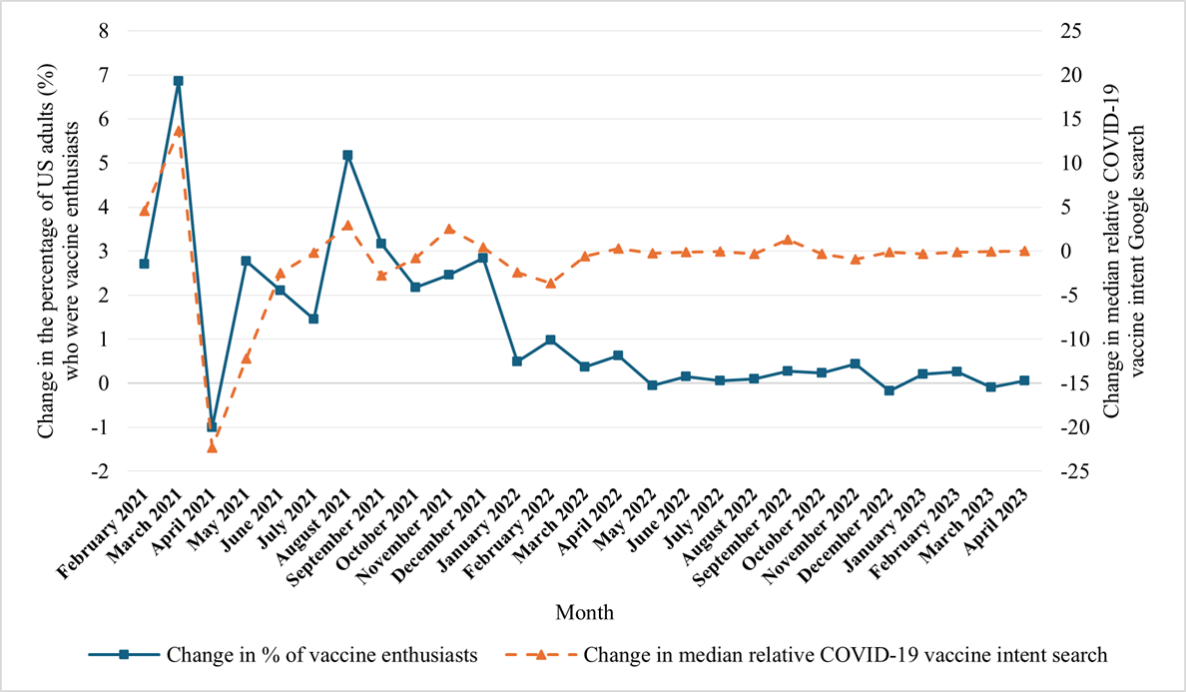
COVID-19 vaccination intent searches and vaccine enthusiasts in the United States (January 2021 to April 2023). The change in the percentage of US adults who were vaccine enthusiasts came from the Monthly Outcome Survey (MOS). Data for the change in the median relative COVID-19 vaccination intent search came from Google’s Vaccination Search Insights index.

Figure 2 illustrates monthly changes in the percentage of US adults who were vaccine enthusiasts between February 2021 and April 2023 [67]. The figure indicates that the percentage of adults who became vaccine enthusiasts increased to varying degrees throughout 2021 in most months. From February to April 2021, for example, vaccine enthusiasts may have performed many vaccine intent searches on Google because it was difficult to find vaccines for them or their families during this period. Thus, it makes sense that there was a strong positive relationship during this period for vaccine enthusiasts but a negative relationship after they got vaccinated.

In addition, this visual shows changes in COVID-19 vaccine intent relative search on Google in 2021 that corresponded with changes in the proportion of adults who became vaccine ready. This suggests that vaccine intent searches could be associated with the first step people made in the vaccination information–seeking process to move from the wait-and-see stage to the vaccine-enthusiast stage. In this respect, vaccine intent searches may serve as a proxy for incident vaccine-ready cases.

Table 1 provides summary statistics for each variable included in the models. For example, in our sample, the mean of the monthly median COVID-19 vaccine intent relative search volume was 7.35 (SD 10.70; range 0.18-111.26); the mean monthly change in vaccine enthusiasts was 0.01 (SD 0.03; range –0.30 to 0.23); and the mean monthly change in the wait-and-see group was –0.01 (SD 0.02; range –0.24 to 0.39). More details about the model covariates are provided in the Data Sources and Analytic Method section of Multimedia Appendix 1.

**Table 1:** Descriptive statistics of the analytic variables in the United States, January 2021 to April 2023.

Variable			Values, n		Values, mean (SD; range)
**Dependent variable**					
	Monthly median COVID-19 vaccine intent relative search volume	14,087		7.350 (10.697; 0.182 to 111.261)	
**Independent variables**					
	Monthly change in vaccine enthusiasts	13,496		0.012 (0.033; –0.304 to 0.231)	
	Monthly change in the wait-and-see group	13,496		–0.011 (0.023; –0.240 to 0.388)	
	Monthly change in the no-intention-to-get-vaccinated group (reference)	13,496		–0.0003 (0.026; –0.201 to 0.217)	
**Health care capacity, internet access, and COVID-19 variables**					
	Mean internet access (raw)	14,087		0.811 (0.125; 0.102 to 1.000)	
	Mean internet access (natural log)	14,087		0.591 (0.076; 0.097 to 0.693)	
	Public health research facilities (raw)	14,087		0.232 (0.879; 0.000 to 11.000)	
	Public health research facilities (natural log)	14,087		0.117 (0.345; 0.000 to 2.485)	
	Access to primary care physicians (raw)	14,087		1.287 (6.033; 0.003 to 124.749)	
	Access to primary care physicians (natural log)	14,087		0.504 (0.559; 0.003 to 4.834)	
	Monthly change in COVID-19 cases per capita_t–1_ (raw)	13,496		0.009 (0.012; 0.000 to 0.128)	
	Monthly change in COVID-19 cases per capita_t–1_ (natural log)	13,496		0.008 (0.010; 0.000 to 0.095)	
	Monthly change in COVID-19 vaccination doses per capita_t–1_ (raw)	13,166		0.030 (0.051; –0.373 to 0.871)	
	Monthly change in COVID-19 vaccination doses per capita_t–1_ (natural log)	13,166		0.021 (0.040; –0.168 to 0.626)	
**Demographics**					
	Income per capita (raw)	14,087		58,032 (16,603; 31,153 to 191,220)	
	Income per capita (natural log)	14,087		10.937 (0.238; 10.347 to 12.161)	
	Change in ideology	14,087		–0.073 (0.053; –0.254 to 0.109)	
	Electoral pivot county	14,087		0.066 (0.248; 0.000 to 1.000)	
	Proportion of non-Hispanic White people (reference)	14,087		0.637 (0.184; 0.036 to 0.920)	
	Proportion of non-Hispanic Black people	14,087		0.111 (0.114; 0.003 to 0.691)	
	Proportion of Hispanic people	14,087		0.150 (0.145; 0.012 to 0.952)	
	Proportion of non-Hispanic Asian American and Pacific Islander people	14,087		0.048 (0.058; 0.004 to 0.559)	
	Proportion of non-Hispanic American Indian and Alaska Native people	14,087		0.007 (0.028; 0.001 to 0.436)	

### Regression Modeling

Table 2 provides the results from the regression model that evaluates the relationship between county-level changes in COVID-19 vaccine readiness and the COVID-19 vaccine intent relative search volume. This model generally performed well, as the overall *R*^2^ value was 0.752. When accounting for variance within the panel, the *R*^2^ value was 0.771, whereas the *R*^2^ value when considering variance between counties was 0.310.

**Table 2:** Effect of county-level COVID-19 vaccine readiness on the COVID-19 vaccine intent relative search volume in the United States (January 2021 to April 2023).^a,b,c^

Variables		Model value (SE)	*P* value
**Independent variables**			
	Monthly change in vaccine enthusiasts	–10.232 (1.289)	<.001
	Monthly change in the wait-and-see group	9.123 (2.540)	<.001
**Health care capacity, internet access, and COVID-19 variables**			
	Mean internet access (natural log)	–2.504 (1.110)	.02
	Public health research facilities (natural log)	0.415 (0.236)	.08
	Access to primary care physicians (natural log)^d^	0.859 (0.263)	.001
	Monthly change in COVID-19 cases per capita_t–1_ (natural log)	124.106 (11.621)	<.001
	Monthly change in COVID-19 vaccination doses per capita_t–1_ (natural log)	25.288 (3.315)	<.001
**Demographics**			
	Income per capita (natural log)	4.856 (0.559)	<.001
	Change in ideology	4.210 (1.473)	.004
	Electoral pivot county	0.553 (0.263)	.04
	Proportion of non-Hispanic Black people	–4.889 (0.796)	<.001
	Proportion of Hispanic people	–1.115 (0.573)	.052
	Proportion of non-Hispanic Asian American and Pacific Islander people	2.210 (2.164)	.31
	Proportion of non-Hispanic American Indian and Alaska Native people	–5.504 (2.334)	.02
	Intercept	–26.945 (6.225)	<.001
**Model statistics**			
	N	13,166	—^e^
	N (counties)	587	—
	*R*^2^ (between)	0.310	—
	*R*^2^ (within)	0.771	—
	*R*^2^ (overall)	0.752	—
	Wald chi-square	20,835.93	<.001

^a^SEs in parentheses are clustered robust SEs.

^b^Coefficients were computed by using a generalized least squares panel regression with time-fixed effects.

^c^Binaries for fixed effects were excluded from this table.

^d^Staffed intensive care beds were perfectly colinear with the number of primary care physicians. Therefore, these 2 variables appeared in separate models.

^e^Not applicable.

The model indicates a negative association between COVID-19 vaccine readiness and the COVID-19 vaccine intent relative search volume: counties with a higher proportion of vaccine enthusiasts relative to other counties were associated with decreased COVID-19 vaccine intent relative search volume across all models (β=–10.232; *Z*=–7.94; *P*<.001). Moreover, counties with a higher proportion of people in the wait-and-see group relative to other counties were associated with increased COVID-19 vaccine intent relative search volume across all models (β=9.123; *Z*=3.59; *P*<.001). The results support our hypotheses of a negative relationship between counties with a higher proportion of vaccine enthusiasts relative to other counties and the COVID-19 vaccine intent relative search volume (hypothesis 1) and a positive relationship between counties with a higher proportion of individuals in the wait-and-see group compared to other counties and the COVID-19 vaccine intent relative search volume compared to other counties (hypothesis 2).

The results demonstrated that several covariates relevant to health care capacity, internet access, and COVID-19 were significantly associated with COVID-19 vaccine intent relative search volume. We found a negative, statistically significant association between internet access and the COVID-19 vaccine intent relative search volume (β=–2.504; *Z*=–2.26; *P*=.02). However, this result was likely an artifact, capturing the effect of population density and economic development on access to COVID-19 pandemic response resources. In the *Robustness Checks Using Varied Operationalizations of the Explanatory Variables* section of Multimedia Appendix 1, we report results from analyses in which we disaggregated mean internet rate by fixed and mobile broadband accesses to elaborate on this effect.

Monthly changes in COVID-19 cases (β=124.106; *Z*=10.68; *P*<.001) and COVID-19 vaccinations administered (β=25.288; *Z*=7.63; *P*<.001) were positively and significantly associated with increased COVID-19 vaccine intent relative search volume. In addition, an increase in access to primary care physicians in a county was associated with increased COVID-19 vaccine intent relative search volume (β=0.859; *Z*=3.27; *P*=.001).

The results reported in Table 2 indicated significant associations between several demographic variables and the COVID-19 vaccine intent relative search volume. Wealthier counties were associated with higher COVID-19 vaccine intent relative search volume (β=4.856; *Z*=8.68; *P*<.001). Counties that became more ideologically conservative (β=4.210; *Z*=2.86; *P*=.004) and electoral pivot counties (β=0.553; *Z*=2.10; *P*=.04) were associated with higher COVID-19 vaccine intent relative search volume. Counties that comprised higher proportions of historically underserved populations, particularly non-Hispanic Black people (β=–4.889; *Z*=–6.14; *P*<.001) and non-Hispanic American Indian and Alaska Native people (β=–5.504; *Z*=2.36; *P*=.02) compared with non-Hispanic White people, were associated with lower COVID-19 vaccine intent relative search volume.

### Results From Predictive Margins Tests

**Figure 3:**
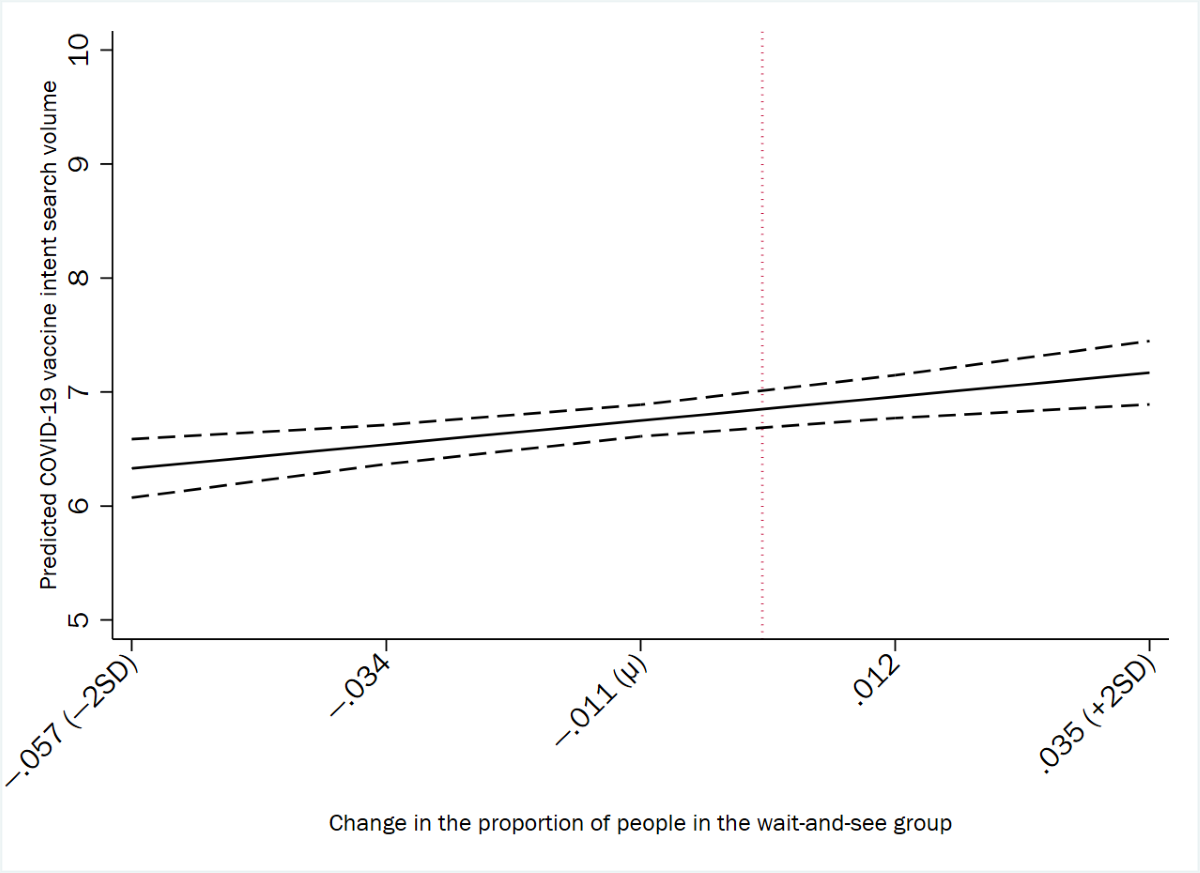
Predicted COVID-19 vaccine intent search volume and the county-level change in the proportion of people in the wait-and-see group with 95% CIs in the United States (January 2021 to April 2023). The vertical reference line represents counties for which there was no change in their wait-and-see proportion between study waves.

**Figure 4:**
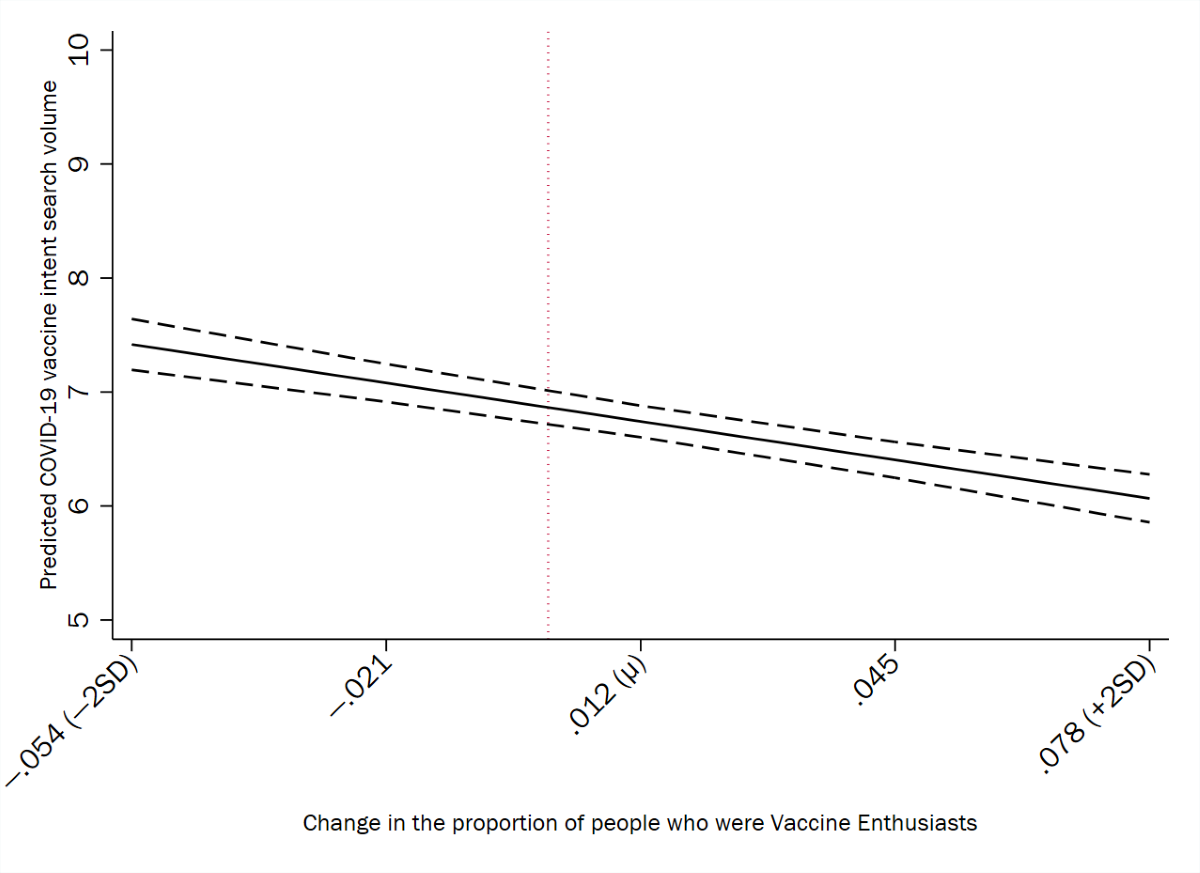
Predicted COVID-19 vaccine intent search volume and the county-level change in the proportion of people who were vaccine enthusiasts with 95% CIs in the United States (January 2021 to April 2023). The vertical reference line represents counties for which there was no change in their vaccine enthusiasm proportion between study waves.

Figures 3 and 4 graphically illustrate the results from the predictive margins tests of county-level vaccine readiness and vaccine intent Google searches in the United States. Figure 3 denotes the predicted COVID-19 vaccine intent search volume that corresponds with county-level changes in the proportion of wait-and-see individuals, whereas Figure 4 represents the predicted COVID-19 vaccine intent search volume that corresponds with county-level changes in the proportion of vaccine enthusiasts.

At the county level, the results from predictive margins tests indicate that changes in the proportion of individuals in the wait-and-see group were positively associated with the COVID-19 vaccine intent relative search volume. Specifically, when the change in the county-level wait-and-see proportion moved from 0 (ie, unchanged counties) to a mean of –0.011 (SD 0.023), this difference was associated with a 1.47% decrease in the predicted county-level COVID-19 vaccine intent relative search volume. An increase in the change in the county-level wait-and-see proportion from 0 to 2 SDs above the mean of 0.035 was associated with a predicted county-level COVID-19 vaccine intent relative search volume of 7.169, reflecting a 4.66% increase from unchanged counties. Finally, when the change in the county-level wait-and-see proportion decreased from 0 to 2 SDs below the mean of –0.057, this difference was associated with a predicted COVID-19 vaccine intent relative search volume of 6.330, reflecting a 7.59% decrease from unchanged counties.

Changes in the county-level vaccine enthusiasm were negatively associated with the COVID-19 vaccine intent relative search volume. Specifically, an increase in the county-level vaccine-enthusiast proportion from 0 to a mean of 0.012 (SD 0.033) was associated with a 1.79% decrease in the predicted county-level COVID-19 vaccine intent relative search volume. An increase in the county-level vaccine-enthusiast proportion from 0 to 2 SDs above the mean of 0.078 was associated with a predicted county-level COVID-19 vaccine intent relative search volume of 6.067, reflecting an 11.63% decrease from unchanged counties. A decrease in the change in the county-level vaccine-enthusiast proportion from 0 to 2 SDs below the mean of –0.054 was associated with a predicted COVID-19 vaccine intent relative search volume of 7.417, reflecting an 8.05% increase from unchanged counties.

### Robustness Checks

We performed several robustness checks to verify whether the observed results were products of the way in which we operationalized our variables. To check whether our results were a consequence of our choice of dependent variable, we used separate models in which we replaced our dependent variable with the mean COVID-19 vaccine intent relative search volume, minimum COVID-19 vaccine intent relative search volume, maximum COVID-19 vaccine intent relative search volume, aggregate COVID-19 vaccine search volumes, and COVID-19 vaccine safety search volumes. To verify whether our results were a consequence of how we operationalized our control variables, we used separate models in which we disaggregated internet access into 2 components, the natural logs of mobile and fixed internet access rates, and included the natural log of the total number of hospitals per county, the natural log of the number of total hospital staff per capita in each county, substituted naturally logged COVID-19 deaths per capita for naturally logged COVID-19 cases per capita, and 2 operationalizations of the CDC Agency for Toxic Substances and Disease Registry social vulnerability index. In general, these robustness checks did not result in substantive differences in the main findings. These checks provided evidence that the modeling was robust to alternative specifications of the dependent and control variables. More details about the robustness checks are available in Multimedia Appendix 1.

## Discussion

### Main Findings

This study examined the relationship between COVID-19 vaccine readiness and the COVID-19 vaccine intent relative search volume on Google. High-level findings are summarized in Table 3. Relative to other counties, counties with higher proportions of vaccine enthusiasts were associated with decreases in county-level COVID-19 vaccine intent relative search volume, and counties with higher proportions of individuals in the wait-and-see group were associated with increases in county-level COVID-19 vaccine intent relative search volume.

**Table 3:** Summary of statistical findings on COVID-19 vaccine intent relative search volume on Google.

Variable	Statistically significant	Direction of effect
Monthly change in vaccine enthusiasts	✓ **(***P*<.001)	–
Monthly change in the wait-and-see group	✓ **(***P*<.001)	**+**

Relative to other counties, counties with a higher proportion of people in the wait-and-see group may have had more individuals who perceived an information gap and used web-based searches to fill this gap, leading to observed increases in vaccine intent searches. This proposed explanation aligns with other research showing that information gaps and vaccine hesitancy were associated with increased information-seeking activity, especially during the time frame of the COVID-19 pandemic that we investigated [13,19,20,37,41]. These results suggest that individuals who were hesitant to receive COVID-19 vaccinations sought information on how to get their COVID-19 vaccination once they made the decision to get vaccinated. When these individuals performed intent-related searches, they likely became vaccine enthusiasts (ie, individuals who were ready to get vaccinated or got a COVID-19 vaccine). In aggregate, their decision to become vaccine enthusiasts helped lower fatality rates from COVID-19. Countries that vaccinated >26 million people substantially decreased their COVID-19 fatality rates from an average of 13% to 1% [27].

Our results further showed that several health care capacity and COVID-19 indicators were associated with higher levels of COVID-19 vaccine intent relative search volume. These increases align with research that has linked changes in COVID-19 cases and vaccinations to subsequent rises in COVID-19 vaccine search [4,41]. Results pertaining to health care capacity suggest that there were differences between access to health care information and access to health care resources as they related to COVID-19 vaccine search.

Our main findings remained statistically significant after accounting for county-level sociodemographic characteristics, indicators of county-level public health infrastructure, COVID-19 cases per capita, and COVID-19 vaccination doses per capita and were robust to alternative specifications of the dependent and independent variables. Consistent with the TPB and PRISM as well as prior research on the association between Google searches and COVID-19 vaccine hesitancy [4,37,41], counties that had a high proportion of vaccine enthusiasts had a smaller proportion of people who perceived limitations in their knowledge. This may explain the observed lower levels of vaccine intent searches in these counties.

In line with the TPB and PRISM and previous research on the relationship between Google search and COVID-19 vaccine hesitancy [4,37,41], it is feasible that increases in the proportion of individuals in the wait-and-see group corresponded with greater perceived limitations in their knowledge about mitigating risks related to COVID-19; these individuals may have sought to address these limitations by performing COVID-19 vaccine searches on Google. Once individuals in the wait-and-see group filled their perceived knowledge gap through vaccine searches, there was a corresponding shift from the decision to wait and see toward vaccine enthusiasm. The information acquired through those earlier searches and exposure to more information on COVID-19 vaccines may have influenced individuals’ beliefs and attitudes toward vaccination. This shift and belief change may have influenced individuals’ decisions to perform vaccine intent searches on Google to gain information about the availability, accessibility, and eligibility of COVID-19 vaccines.

In addition, the internet can act as a valuable information source for supporting health decision-making and other health behaviors. Our study results highlight the importance of having available, actionable, clear, and credible information about health behaviors, as people are more likely to seek health information from the internet when this information is available, clear, actionable, and credible [11,12]. Public health researchers and practitioners may benefit from additional research that queries the extent to which the decision-making processes that we have modeled operate when examining other vaccine-preventable diseases such as influenza and Mpox.

Our findings complement existing research on the effects of information seeking on public health, as we found that a higher county-level proportion of individuals in the wait-and-see group was associated with increases in county-level search volume for COVID-19 vaccine intent. These findings build upon other research that finds a negative association between searches for both “COVID anxiety” and “COVID depression” and the total number of vaccinations between August 2020 and November 2021 [32]. Similarly, there was an initial spike in searches related to the unfounded link between infertility and receiving a COVID-19 vaccine right after the emergency use authorization [38,39], followed by a marked decrease in the volume of these searches as people received COVID-19 vaccinations [40]. As more members of the wait-and-see group became vaccinated in the United States, their decision to become vaccinated helped lower fatality rates from COVID-19 [27]. Thus, increases in county-level proportions of people who became vaccine enthusiasts were associated with decreased county-level search volumes for COVID-19 vaccine intent, COVID anxiety, COVID depression, and infertility as a result of receiving a COVID-19 vaccine.

Moreover, our results suggest that some of the people who most needed access to this information to reduce perceived knowledge gaps about COVID-19 vaccination sought higher volumes of this information than vaccine enthusiasts or members of the no-intention-to-get-vaccinated group. Finally, this research underscores the potential for the use of Google and other web search data as early indicators of or proxies for subsequent health-related behaviors, making them valuable tools for public health efforts or interventions that seek to understand and promote vaccination and other health-related behaviors. Public health interventions or efforts can also use web search data to inform their public education efforts during evolving public health crises in a way that furnishes the information that the public needs to make health decisions.

### Limitations

Although the findings from this research present compelling insights, we acknowledge several study limitations. First, Google’s reporting of its search metrics was not uniform across data points. Specifically, artificial noise was intentionally added to data points to safeguard user privacy [43,59]. Although the presence of artificial noise in these data introduced some bias, this bias is nonsystematic in nature [59]. Nonsystematic measurement error could influence the certainty of our conclusions, but it did not introduce any systematic bias into the findings [68].

Second, it is possible that our model estimates did not sufficiently consider uneven variance in the COVID-19 vaccine intent relative search volume across counties. To account for uneven variance, we clustered the SEs by county [65]. However, this is a conservative approach to estimating SEs, as any remaining bias in statistical significance tests is likely against obtaining statistically significant findings, as the SEs may be too large [65]. Other approaches to estimating SEs may more effectively account for this uneven variance.

Third, the hypotheses that we tested were specific to the time frame that we examined. At the beginning of this time frame, COVID-19 vaccines were newly introduced to the population and becoming more widely available in the United States. Fourth, there are several limitations to this study related to the MOS small area estimates. These estimates relied on self-reported data from respondents with respect to vaccine readiness. Consequently, this measure may be subject to issues such as recall bias, selective recall, respondent bias, and social desirability bias [69]. In addition, those who solely or primarily speak languages other than English or Spanish are likely underrepresented in these estimates, as the MOS surveys were administered only in English or Spanish.

Fifth, there are 2 limitations related to web-based search. Google search does not represent all web-based searches. Although unlikely, given Google’s dominant market share in the United States, it is possible that people varied their search behavior by platform, using some platforms for selected searches while others for different searches. In a similar vein, web-based search is not the only form of information seeking, as people seek health information from many sources, including medical providers [5] and traditional media sources such as broadcast television and radio [6].

### Conclusions

Internet-based intent search presents an important signal for vaccine readiness change. During this period of steep increase in the uptake of primary COVID-19 vaccine series [42], the volume of vaccine intent searches was high in high wait-and-see counties and less in counties with high levels of vaccine enthusiasts. Considering that previous findings identified a relationship between vaccine intent search and subsequent vaccine uptake, these findings may indicate that vaccine intent searches aligned with people’s transition from the wait-and-see stage to the vaccine-enthusiast stage [41]. Our findings build upon a growing body of literature and indicate an association between changes in COVID-19 vaccine readiness among adults and increased COVID-19 vaccine intent relative search volume. These results reinforce the promise of using search data as a signal, an early measure, or a proxy for subsequent health behaviors and navigate methods to jointly use search and survey data, using web-based search as a proxy for information seeking and decision-making to assess the association of these behavioral precursors and readiness *with* offline health behavior.

Because Google search plays an important role for those in the planning phase before they commit to or act upon health-related behaviors, these results have implications for public health campaign interventions. Google search data can be used to measure public health intervention or effort effectiveness and track the spread of misinformation or hesitancy about health-related behaviors [40,70]. As search trends can often be identified more quickly than self-reported behaviors or beliefs in surveys, this provides an opportunity for more timely strategic pivots by public health interventions or efforts. Thus, Google search data can be used to optimize the use of campaign resources such as paid media advertising and distribution.

Those who undertake public health interventions may provide additional, tailored information to educate the public in the areas in which there may be a higher proportion of those who are hesitant (ie, wait and see) but open to performing a particular positive health behavior (eg, vaccination), as this information can catalyze their decision-making and information-seeking processes toward that behavior. Public health education interventions can also include employer-based vaccine promotion outreach, as evidence indicates that working in organizations with higher perceived COVID-19 safety climates was associated with subsequent increases in vaccine readiness [71].

Similarly, public health interventions can allocate specific, tailored resources to promote positive health behaviors, including establishing additional clinics in selected locales and enhancing local internet infrastructure. To encourage vaccination, especially for children, these resources should be widely accessible with the least possible financial burden on individuals, families, and communities [72]. These resources can make it easier for people to perform health behavior intent searches and follow through with those positive health behaviors as soon as possible.

These results also have implications for public policy and public policy makers because vaccinations are a necessary, but by themselves insufficient, public policy solution to pandemics [25,26,73-76]. Vaccinations are one piece of the broader health policy landscape because there is an upper limit of a population that is vaccinable without encountering vaccine hesitancy [74,75]. While it is possible that vaccination can lead to decreased risk perception, which is postulated in the Peltzman effect [75,77], other literature shows that those who were COVID-19 vaccinated in the United States were also more likely to engage in other COVID-19 prevention behaviors such as mask wearing and social distancing [78]. It is important to use a holistic approach to assess the effectiveness of individual-level measures to modify behavior.

Beyond vaccination distribution and promotion, other country-level measures are associated with improved public health outcomes during pandemics [74]. These include having better early detection systems tracking disease cases [73], improved medical recordkeeping [25], and more resources and support for rapid vaccine development and dissemination [73]. More broadly, countries that had a higher gross domestic product per capita [74], high average levels of health care expenses overall [24], lower levels of air pollution [75], and more effective public governance structures [76] were associated with higher COVID-19 vaccination uptake and fewer negative effects from the COVID-19 pandemic. During pandemics, countries that carefully monitor vaccine intent search data can use data-centric strategies to their benefit. We found a positive association between the county-level proportion of unvaccinated adults who indicated that they would wait and see before getting a COVID-19 vaccine and COVID-19 vaccine intent relative search volume.

## Appendix

Multimedia Appendix 1 Additional analyses and description of our analytic procedures.

## Abbreviations

AHRF: Area Health Resources File
CDC: Centers for Disease Control and Prevention
FCC: Federal Communications Commission
HHS: Department of Health and Human Services
MOS: Monthly Outcome Survey
PRISM: Planned Risk Information Seeking Model
TPB: theory of planned behavior

## Data Availability

The data sets generated during and analyzed during this study are available from several sources including Google’s COVID-19 Vaccination Search Insights database [43], the US Department of Health and Human Services’ (HHS) health database [47], the US Centers for Disease Control and Prevention’s Weekly United States COVID-19 Cases and Deaths by County database [48], the HHS Area Health Resources Files [49], the US Federal Communications Commission’s Form 477 County Data on Internet Access Services [50], and Warshaw and Tausanovitch’s [51] Harvard Dataverse for the American Ideology Project.
