# Supplementary material for: Analyzing Google COVID-19 Vaccine Intent Search Trends and Vaccine Readiness in the United States: Panel Data Study
Source: Online J Public Health Inform. 2024 Jul 29;16:e55422. doi: 10.2196/55422 (PMC11319879; doi:10.2196/55422)
Supplement: Multimedia Appendix 1 [file ojphi_v16i1e55422_app1.docx]

**Multimedia Appendix 1 for *Analyzing Google COVID-19 Vaccine Intent Search Trends and Vaccine Readiness in the United States: Panel Data Study***

## Data sources and analytic method

In this section, we provide additional details about three of the data sources, how we prepared the data for analysis, and our analytic method.

### Google Vaccine Search Insights data

We used Google Vaccine Search Insights data to operationalize behavior surrounding the COVID-19 vaccine. Google updated these data weekly and included county and ZIP code-level information. Google aggregated and anonymized data to understand and compare communities’ information needs while protecting the privacy of its users [59]. These data illustrated people’s interest in COVID-19 vaccines and other relevant topics by gauging search volume and were not meant to measure opinions or actual events surrounding the topic of COVID-19 vaccines. This publicly available data set has been used in research and evaluation of COVID-19 vaccine behavior [37, 41, 53].

The COVID-19 vaccine intent search measure that we used represents relative interest in COVID-19 vaccine compared to all other search topics, scaled by Google’s methods, to easily compare regions over time or to one other [43]. This measure included content related to COVID-19 vaccine availability, accessibility, and eligibility [43]. We used the median COVID-19 vaccine intent relative search volume for each county in each month to aggregate these data. The median was used because it more effectively considered potential skewness in the distribution of values for each county versus the mean. As a robustness check on the results, we replaced the median COVID-19 vaccine intent relative search volume with the mean, minimum, and maximum values of the COVID-19 vaccine intent relative search volume for each county in each month. This robustness check is provided in the “Robustness checks using varied operationalizations of the dependent variable” section, Table S3.

The data were limited to comply with Google’s required privacy protocols for all search data. For example, Google excluded individual-level searches or access to personally identifiable information (PII) for users [59]. Instead, Google provided aggregate-level data at the ZIP code level with artificial, nonsystematic noise added to provide differential privacy to users or groups of users who could be reidentified [43,59]. Additionally, there were data gaps due to the lack of ZIP code data for regions that were smaller than 3 square kilometers or had a population under 100,000 people [43]. To better suppress potential breach of user data, Google used a proprietary differential privacy algorithm to add artificial noise to data points included in the data set [43,59]. Google assessed the size of the signal (i.e., the actual count of queries in each category, place, and time). Once the impact of the signal was assessed, Google determined whether data should be reported for that category or whether the data should be excluded for being too heavily noised [43,59]. For more information on Google’s approach, please visit Google’s COVID-19 Vaccination Search Insights page [43,59].

### Monthly Outcome Survey data

The Monthly Outcome Survey (MOS) provided data on vaccine readiness. These publicly available data come from a series of cross-sectional, nationally representative surveys that were administered monthly between January 2021 (Wave 1) and April 2023 (Wave 28) by the U.S. Department of Health and Human Services (HHS) [67]. Fors Marsh led the study and fielded all survey waves in its role as the prime contractor for the HHS COVID-19 public education campaign. Among other items, the MOS assessed COVID-19 vaccine readiness and uptake. The monthly sample size for each wave was approximately 5,000 adults and came from Ipsos’ KnowledgePanel, an online, nationally representative, probability-based panel of adults in the United States (U.S.) [67]. All participants provided written informed consent prior to participating in the survey.

Small area estimates were derived from the MOS to construct county-level estimates of the proportions of the three vaccine readiness groups: Vaccine Enthusiasts, Wait and See, and No Intention to Vaccinate. A gradient booster classifier model produced predicted probabilities of each of the three levels of vaccine readiness across age, sex, and education census tract subgroups [47]. Beyond this demographic information, geographic characteristics (like population density and rurality or urbanicity) and indicators of political leaning (including county-level vote shares for presidential candidates in the 2016 and 2020 general elections) were also incorporated into the model [47]. Then, an iterative proportional fitting algorithm was applied to these estimates to ensure they were consistent with the MOS’s U.S. national estimates given weighting by population size [47,54].

We examined the correlations between both county-level group estimates and potential demographic covariates to determine which ones to include in the models that appear in Table 2 in the manuscript and which ones should be analyzed separately. Potential demographic covariates with variance inflation factors (VIF) of less than 2 were included into the manuscript models. In addition, potential demographic covariates with correlations less than +/- 0.25 were included in the manuscript models [55]. These covariates included measures of income per capita and race and ethnicity.

When we employed these decision rules, values for each of these county-level estimates were not sufficiently correlated with county level per capita covariates sourced from the Area Health Resources Files (AHRF), including race and ethnicity [49]. The correlations for the natural log of mean internet access and each county-level estimate that we used were 0.141 (Vaccine Enthusiasm) and -0.082 (Wait and See). In addition, the correlations between race and ethnicity ranged from -0.106 (proportion who are non-Hispanic Asian American and Pacific Islander people and Wait and See) to 0.263 (proportion who are non-Hispanic Asian American and Pacific Islander people and Vaccine Enthusiasts). Table S2 in this Appendix contains estimates of models that excluded these variables. The signs and significance patterns of our primary independent variables (Change in Vaccine Enthusiasts and Change in Wait and See) in the manuscript did not change when aggregate-level covariates for income per capita and race and ethnicity were excluded, thus justifying their inclusion into the models contained in the manuscript [47,55]. For more information about the small area estimates, please visit the HHS health data page [47].

### County-level political context data

In the manuscript, we used two indicators that captured political context. The first measure (electoral pivot county) was a binary variable for those counties that voted for the Republican presidential candidate in the 2016 general election and voted for the Democratic presidential candidate in the 2020 general election. One county that met this condition was Maricopa County, Arizona, because the plurality of voters in that county voted for the Republican presidential candidate in 2016 but for the Democratic presidential candidate in 2020.

Our second measure (change in ideology) is based on a measure developed by Tausanovitch and Warshaw [51,52] and was operationalized as the change in county-level political ideology from 2016 to 2020. This measure began with survey data from the Cooperative Election Studies and the UCLA Nationscape Survey to provide initial estimates of ideology [51,52]. These estimates were adjusted by race, education, and gender through a Bayesian item response theory (IRT) model paired with a multilevel regression with poststratification (MRP) model to generate the mean preferences of geographic constituencies [51,52]. Then, these estimates were aggregated to produce estimates of the public’s preferences in each county. For our analyses, we differenced this ideological measure from 2020 to 2016 for each county. Higher values for this measure indicate more conservative counties from 2020 to 2016. For more information about the county-level political context data, please visit the Harvard Dataverse page on which these data are deposited [51].

### Analytic method

In Table 2 of the manuscript, we estimated two random effects generalized least squares regression models with time-fixed effects and clustered robust standard errors by county. The inclusion of time-fixed effects allowed us to unearth statistically significant relationships between our dependent and independent variables while considering the effects of time. The inclusion of fixed effects in these models enabled us to consider the potential effects of external, time-varying factors on COVID-19 vaccine intent relative search volume. For example, many people searched for information about COVID-19 vaccination when vaccines were first publicly available, from January to April 2021 [56]. Search activity fell in the interim periods [43], and then increased during the Delta and Omicron surges later in 2021 and into periods of 2022 [57]. We clustered the standard errors by county to account for uneven variance in COVID-19 vaccine intent relative search volume across counties.

After estimating our models, we performed predictive margins tests to calculate more precise estimates of the substantive impact of both COVID-19 vaccine readiness variables on COVID-19 vaccine intent relative search volume. These estimates were executed because regression model coefficients provide linear point estimates that neither fully capture the statistical effects being modeled nor add the appropriate nuances to the statistical results. We used percentage changes in these tests since the numeric values for our dependent variable have no straightforward, intuitive meaning. Each result from a predictive margins test furnished the percentage change in the value of the dependent variable given a specified change in the value of a single independent variable. To perform these tests, we held all control variables at their means while changing the value of each of our primary independent variables, one at a time, from their respective mean values to up to 2 standard deviations away from those mean values (μ). We employed 2 standard deviations away from the mean to illustrate what happens when counties vary widely with respect to the Vaccine Enthusiast and Wait and See groups.

## Descriptive statistics for supplemental variables

In this section, we provide descriptive statistics and measurement notes for variables that appeared in the Multimedia Appendix but not in the manuscript. These include varied operationalizations of the dependent and independent variables.

### Alternative dependent variables

We tested five different operationalizations of the dependent variable for which we provide summary statistics in Table S1. These operationalizations were subdivided into two groups: (1) variables that measured the content of Google search behavior, and (2) variables that measured the volume of Google search behavior relative to the county level.

Google used content analyses to classify COVID-19 vaccines searches into *vaccine intent* searches, *vaccine safety* searches, and *aggregate vaccine* searches. Despite this classification, overlap existed across the categories, suggesting that searchers queried multiple topics simultaneously or concurrently. We focused on intent in the manuscript, which was associated with words and phrases for those seeking vaccination. This type of search could theoretically be connected to a probabilistic change in behavior. As a robustness check on the results in Table 2 of the manuscript, we ran our model with information-seeking search behavior, including *vaccine safety* and *aggregate vaccine* searches. Those models are provided in Table S4 in of the “Robustness checks using varied operationalizations of the dependent variable” section.

Google used a scaling methodology to determine the relative volume of vaccine-oriented searches at different levels of analysis. To consider the noise that Google added to the data, we collapsed weekly ZIP code-level searches into monthly, county-level median search volumes. As robustness checks, we substituted the median measure used in Table 2 of the manuscript with the mean, minimum, and maximum COVID-19 vaccine intent relative search volumes at the county- level. The models with those substituted dependent variables are provided in Table S3 of the “Robustness checks using varied operationalizations of the dependent variable” section.

### Alternate covariates

Consistent with the main analyses in Table 2 of the manuscript, alternate covariates in the Multimedia Appendix are categorized into two groups: (1) health care capacity, internet access, and COVID-19 variables; and (2) demographic characteristics. One unanticipated finding from the models contained in Table 2 in the manuscript was the relationship between internet access and vaccine search behavior, so we disaggregated internet access into its constituent parts: the rate of access to fixed internet and the rate of access to mobile broadband 4G coverage. The models with this disaggregation are available in Table S5 of the “Robustness checks using varied operationalizations of the explanatory variables” section.

The descriptive statistics (Table S1) indicate that counties had higher average levels of mobile broadband access than fixed internet. Maximum fixed internet access was 58.3% county coverage whereas the maximum for mobile broadband was 100% coverage. Further, minimum mobile broadband access was 20.4% coverage whereas minimum fixed internet access was 0% coverage. These data were accessed using the Federal Communications Commission (FCC) 477 County Data on Internet Access Services [50].

Table S1: Descriptive statistics for alternative county-level analytic variables in the U.S., January 2021 to April 2023.^a, b^

|  | **Variable** | **N** | **Mean** | **Standard deviation** | **Minimum** | **Maximum** |
| --- | --- | --- | --- | --- | --- | --- |
| ***Content-oriented dependent variables*** | | | | | | |
|  | Monthly COVID-19 vaccine intent relative search volume (main dependent variable) | 14,087 | 7.350 | 10.697 | 0.182 | 111.261 |
|  | Monthly COVID-19 vaccine safety relative search volume | 15,358 | 4.522 | 5.095 | 0.278 | 40.007 |
|  | Monthly COVID-19 aggregate vaccine relative search volume | 16,527 | 24.453 | 23.832 | 1.595 | 184.442 |
| ***Volume-oriented dependent variables*** | | | | | | |
|  | Monthly mean COVID-19 vaccine intent relative search volume | 14,087 | 8.045 | 11.279 | 0.187 | 109.142 |
|  | Monthly minimum COVID-19 vaccine intent relative search volume | 14,087 | 3.539 | 4.867 | 0.118 | 58.453 |
|  | Monthly maximum COVID-19 vaccine intent relative search volume | 14,087 | 16.385 | 24.107 | 0.199 | 253.092 |
| ***Health care capacity, internet access, and COVID-19 variables*** | | | | | | |
|  | Fixed internet access rate (raw) | 16,527 | 0.367 | 0.083 | 0.000 | 0.583 |
|  | Fixed internet access rate (natural log) | 16,527 | 0.310 | 0.068 | 0.000 | 0.459 |
|  | Mobile broadband internet access rate (raw) | 16,527 | 0.894 | 0.169 | 0.204 | 1.000 |
|  | Mobile broadband internet access rate (natural log) | 16,527 | 0.634 | 0.099 | 0.186 | 0.693 |
|  | Staffed intensive care beds (raw) | 14,087 | 194.987 | 317.567 | 0.000 | 2,378 |
|  | Staffed intensive care beds (natural log) | 14,087 | 4.174 | 1.799 | 0.000 | 7.774 |
|  | Hospital personnel per capita (raw) | 16,527 | 0.0003 | 0.0003 | 0.000 | 0.004 |
|  | Hospital personnel per capita (natural log) | 16,527 | 0.0003 | 0.0003 | 0.000 | 0.004 |
|  | Number of hospitals (raw) | 16,527 | 6.079 | 7.858 | 0.000 | 100.000 |
|  | Number of hospitals (natural log) | 16,527 | 1.663 | 0.706 | 0.000 | 4.615 |
|  | Monthly change in COVID-19 deaths per capita_t – 1_ (raw) | 15,936 | 7.61e-05 | 9.45e-05 | 0.000 | 0.001 |
|  | Monthly change in COVID-19 deaths per capita_t – 1_ (natural log) | 15,936 | 7.59e-05 | 9.42e-05 | 0.000 | 0.001 |
| ***Demographics*** | |  |  |  |  |  |
|  | Social Vulnerability Index | 16,527 | 0.521 | 0.263 | 0.005 | 0.996 |
|  | High-risk county | 16,527 | 0.230 | 0.421 | 0.000 | 1.000 |

^a^ The main dependent variable was included for reference.

^b^ The sample was limited to those cases in which COVID-19 vaccine intent relative search volume was not missing.

Table S1 also provides summary statistics for alternate measures of health care infrastructure, preparation, and overall capacity, including total hospitals, hospital personnel per capita using HHS AHRF [49], and COVID-19 deaths per capita from the Centers for Disease Control and Prevention (CDC) [48]. *Total hospitals (natural log)* measured health care infrastructure, capacity, and coverage at the county level. The descriptive statistics (Table S1) indicate that the mean total hospitals was 6.079 (SD 7.858) hospitals per county, with some having 0 and others having 100. *Hospital personnel per capita* furnished an additional measure of health care capacity, preparation, and coverage by calculating the total number of doctors, nurses, technicians, and administrative staff divided by the total county population. Lastly, *COVID-19 deaths per capita* measured monthly differences in fatalities attributed to COVID-19 infection, controlling for both health care capacity and the progression of COVID-19, as well as the incentive structure to receive a vaccine.

Finally, we examined two alternate measures of income and social inequality at the county level, sourced from the CDC and Agency for Toxic Substances and Disease Registry’s (ATSDR) Social Vulnerability Index (SVI): (1) a continuous measure of the SVI, and (2) a binary variable categorizing the most at-risk counties (high-risk county, henceforth). Both measures provide data on populations who are “especially at risk during public health emergencies because of factors like socioeconomic status, household characteristics, racial and ethnic minority status, or housing type and transportation” [58]. We examined social vulnerability because factors beyond race and ethnicity and income affect a community’s ability to handle and recover from public health emergencies [58].

The SVI is a continuous scaled variable based on answers to 16 U.S. Census Bureau survey items that include measures related to socioeconomic status, household characteristics, housing type and transportation, and racial and ethnic minority status [58]. We used this continuous SVI variable to create the binary high-risk county measure, which indicates counties with the most at-risk populations during an emergency event or disaster. Counties with a score of 0.75 or higher were considered high risk. A county that has a score of 0.75 means that it is in the 75th percentile for social vulnerability relative to other counties in the U.S [58]. The CDC has used this as a threshold for highly vulnerable counties [58]. Counties that met this condition were coded 1 and counties that did not satisfy this criterion were coded 0. The descriptive statistics (Table S1) indicate that the mean of the continuous SVI was 0.521 (SD 0.263), suggesting that the mean county was at the medium to high level of risk. Yet, the high-risk county binary variable had a mean of 0.230 (SD 0.421), suggesting that most of the sample was not at the highest risk of vulnerability.

## Stepwise regression results

In Table 2 of the manuscript, we employed a stepwise approach to estimate both models. This approach provided an internal consistency check on the main findings with respect to including or excluding sets of control variables. The stepwise models are reported in Table S2. Model 1 (the baseline model) estimated the relationship between changes in vaccine readiness and COVID-19 vaccine intent relative search volume with no control variables. In Model 2, we estimated the baseline model and incorporated controls for health care capacity, internet access, and COVID-19 variables, but excluded demographic controls. In Model 3, we estimated the baseline model and only included controls for demographics. In Model 4, we include all variables with one exception: we substituted the natural log of staffed intensive care beds in place of the natural log of access to primary care physicians. These variables were separated due to collinearity. We reported the model that included the natural log of access to primary care physicians in Table 2 of the manuscript because it performed slightly better than Model 4 in Table S2 of this document.

The R^2^ values for these models vary, as including additional independent variables increases the explanatory power of the model. In addition, the demographic covariates appear to have helped the explanatory power of the model when we examine variation between counties. The stepwise models show that the main findings that were reported in the manuscript were not an artifact of the way in which we specified our models, as the signs and significance patterns of both independent variables are identical across the models in Table S2.

Table S2: Effect of county-level COVID-19 vaccine readiness on COVID-19 vaccine intent relative search volume in the U.S., January 2021 to April 2023.^a, b, c^

| **Independent variables** | | **Model 1**  **Value** | ***P* value** | **Model 2**  **Value** | ***P* value** | **Model 3**  **Value** | ***P* value** | **Model 4**  **Value** | ***P* value** |
| --- | --- | --- | --- | --- | --- | --- | --- | --- | --- |
|  | Monthly Change in Vaccine Enthusiasts | -9.153 (1.216) | <.001 | -10.488 (1.286) | <.001 | -9.074 (1.225) | <.001 | -10.209 (1.290) | <.001 |
|  | Monthly Change in Wait and See group | 7.234 (2.450) | .003 | 8.935 (2.601) | .001 | 7.212 (2.390) | .003 | 9.159 (2.550) | <.001 |
|  | Mean internet access | n/a |  | -3.960 (1.547) | .010 | n/a |  | -2.318 (1.093) | .034 |
|  | Public health research facilities (natural log) | n/a |  | 1.786 (0.315) | <.001 | n/a |  | 0.667 (0.248) | .007 |
|  | Staffed intensive care beds (natural log) | n/a |  | 0.080 (0.054) | 0.138 | n/a |  | 0.010 (0.045) | .823 |
|  | Monthly change in COVID-19 cases per capita_t – 1_ (natural log) | n/a |  | 124.441 (11.800) | <.001 | n/a |  | 126.421 (11.614) | <.001 |
|  | Monthly change in COVID-19 vaccination doses per capita_t - 1_ (natural log) | n/a |  | 25.783 (3.414) | <.001 | n/a |  | 25.406 (3.323) | <.001 |
|  | Income per capita (natural log) | n/a |  | n/a |  | 6.156 (0.507) | <.001 | 5.554 (0.552) | <.001 |
|  | Change in ideology | n/a |  | n/a |  | 4.859 (1.460) | .001 | 4.432 (1.467) | .003 |
|  | Electoral pivot county | n/a |  | n/a |  | 0.649 (0.251) | .010 | 0.534 (0.263) | .043 |
|  | Proportion who are non-Hispanic Black people | n/a |  | n/a |  | -3.166 (0.657) | <.001 | -3.651 (0.715) | <.001 |
|  | Proportion who are Hispanic people | n/a |  | n/a |  | -0.021 (0.566) | .971 | -.809 (0.602) | .179 |
|  | Proportion who are non-Hispanic Asian American and Pacific Islander people | n/a |  | n/a |  | 4.150 (1.819) | .023 | 5.133 (2.239) | .022 |
|  | Proportion who are non-Hispanic American Indian and Alaska Native People | n/a |  | n/a |  | -3.759 (2.452) | .125 | -5.611 (2.544) | .027 |
|  | Intercept | 26.363 (0.651) | <.001 | 25.904 (1.106) | <.001 | -40.345  (5.498) | <.001 | -34.656 (6.157) | <.001 |
|  | *N* | 13,496 |  | 13,166 |  | 13,496 |  | 13,166 |  |
|  | *N* (Counties) | 591 |  | 587 |  | 591 |  | 587 |  |
|  | *R*^2^ (between) | 0.002 |  | 0.006 |  | 0.247 |  | 0.290 |  |
|  | *R*^2^ (within) | 0.761 |  | 0.771 |  | 0.762 |  | 0.771 |  |
|  | *R*^2^ (overall) | 0.713 |  | 0.728 |  | 0.740 |  | 0.751 |  |
|  | Wald Chi-Square | 21,851.61 | <.001 | 20,646.85 | <.001 | 20,416.99 | <.001 | 20,555.93 | <.001 |

^a^ Standard errors in parenthesis are clustered robust standard errors (CRSE).

^b^Coefficients were computed by using a generalized least squares (GLS) panel regression with time-fixed effects.

^c^ Binaries for fixed effects were excluded from this table.

## Robustness checks using varied operationalizations of the dependent variable

### Mean, minimum, and maximum COVID-19 vaccine intent

The model summarized in Table 2 in the manuscript used median COVID-19 vaccine intent relative search volumes as the dependent variable. One limitation of that analysis is that using median intent may not fully capture the statistical properties of what happens with respect to COVID-19 vaccine intent relative search volume. The median takes the middle value of a collection of values and may not represent the underlying distribution of the data for each county because of the aggregation that occurred to compute it. To consider the distributional properties of this variable more fully across counties, we substituted our dependent variable with three variants: mean COVID-19 vaccine intent relative search volume, minimum COVID-19 vaccine intent relative search volume, and maximum COVID-19 vaccine intent relative search volume. These results are summarized in Table S3.

Following our analyses in Table 2 of the manuscript, we ran separate models for staffed intensive care beds and access to primary care physicians due to collinearity. The remaining robustness checks and alternative specifications in this Multimedia Appendix are based on the model in Table 2 of the manuscript. To recall, this model included access to primary care physicians and excluded staffed intensive care beds due to collinearity. The results were substantially similar to those models where we included staffed intensive care beds but excluded access to primary care physicians, as there were no differences when we examined main findings, and only minor differences on the remaining variables.

These analyses addressed this concern about distributional properties because we used multiple values that came from the same underlying variable distribution, including the lowest, highest, and mean values. Although we did not anticipate any changes in the results when we substituted the mean, changes were possible in the minimum COVID-19 vaccine intent relative search volume and maximum COVID-19 vaccine intent relative search volume values because counties in the U.S. varied widely.

Table S3: Robustness checks using varied operationalizations of COVID-19 vaccine intent relative search volume while omitting access to staffed intensive care beds in the U.S., January 2021 to April 2023.^a, b, c^

| **Independent Variables** | | **Mean Intent**  **Value** | ***P* value** | **Minimum Intent Value** | ***P* value** | **Maximum Intent Value** | ***P* value** |
| --- | --- | --- | --- | --- | --- | --- | --- |
|  | Monthly change in Vaccine Enthusiasts | -7.953 (1.235) | <.001 | -3.759 (.683) | <.001 | -1.547 (2.913) | .595 |
|  | Monthly change in Wait and See group | 8.987 (2.497) | <.001 | 1.724 (1.280) | .178 | 11.618 (5.280) | .028 |
| ***Health care capacity, internet access, and COVID-19 variables*** | | | | | | | |
|  | Mean internet access (natural log) | -2.522 (1.156) | .029 | -0.621 (0.639) | .331 | -4.535 (2.574) | .078 |
|  | Public health facilities (natural log) | 0.549 (0.247) | .026 | 0.030 (0.141) | .832 | 2.291 (.583) | <.001 |
|  | Access to primary care physicians (natural log) | 0.878 (0.268) | .001 | 0.282 (0.106) | .008 | 2.137 (.733) | .004 |
|  | Monthly change in COVID-19 cases per capita_t – 1_ (natural log) | 128.464 (10.824) | <.001 | 58.341 (4.845) | <.001 | 286.299 (22.265) | <.001 |
|  | Monthly change in COVID-19 vaccination doses per capita_t – 1_ (natural log) | 28.341 (3.668) | <.001 | 10.923 (1.502) | <.001 | 62.981 (8.128) | <.001 |
| ***Demographics*** | | | | | | | |
|  | Income per capita (natural log) | 5.000 (0.576) | <.001 | 1.771 (0.266) | <.001 | 9.828 (1.249) | <.001 |
|  | Change in ideology | 4.232 (1.522) | .005 | 1.482 (0.786) | .059 | 8.624 (3.361) | .010 |
|  | Electoral pivot county | 0.513 (0.271) | .059 | 0.376 (0.153) | .014 | 0.760 (0.578) | .189 |
|  | Proportion who are Non-Hispanic Black people | -4.801 (0.803) | <.001 | -3.304 (0.454) | <.001 | -6.388 (1.681) | <.001 |
|  | Proportion who are Hispanic people | -0.932 (0.582) | .109 | -1.455 (0.292) | <.001 | 2.195 (1.219) | .072 |
|  | Proportion who are Non-Hispanic Asian American and Pacific Islander people | 2.355 (2.202) | .285 | 1.780 (1.033) | .085 | 7.642 (5.235) | .144 |
|  | Proportion who are Non-Hispanic American Indian and Alaska Native people | -7.202  (2.553) | .005 | -4.488 (1.219) | <.001 | -14.631 (5.192) | .005 |
|  | Intercept | -27.671 (6.399) | <.001 | -8.086 (2.966) | .006 | -57.666 (13.924) | <.001 |
|  | *N* | 13,166 |  | 13,166 |  | 13,166 |  |
|  | *N*(Counties) | 587 |  | 587 |  | 587 |  |
|  | *R*^2^ (between) | 0.306 |  | 0.269 |  | 0.237 |  |
|  | *R*^2^ (within) | 0.785 |  | 0.747 |  | 0.766 |  |
|  | *R*^2^ (overall) | 0.765 |  | 0.723 |  | 0.743 |  |
|  | Wald Chi-Square | 43,378.52 | <.001 | 9,295.60 | <.001 | 28,118.77 | <.001 |

^a^ Standard errors in parenthesis are clustered robust standard errors (CRSE).

^b^ Coefficients were computed by using a generalized least squares (GLS) panel regression with time-fixed effects.

^c^ Binaries for fixed effects were excluded from this table.

When we performed these analyses, change in Vaccine Enthusiasts had the expected negative sign in all instances and was statistically significant for mean (β = -7.953, Z = -6.44, *P*< .001) and minimum intent (β = -3.759, Z = -5.50, *P*< .001). Moreover, change in Wait and See had the anticipated positive sign in all instances and was statistically significant for mean intent (β = 8.987, Z = 3.60, *P*< .001) and maximum intent in both tables (β = 11.618, Z = 2.20, *P* = .028). In two-thirds of instances, changing how we measured our dependent variable did not affect the sign or significance of the relevant coefficients.

### Aggregate COVID-19 vaccine search and vaccine safety search volumes on Google

The model in Table 2 of the manuscript used COVID-19 vaccine intent relative to search volume as the dependent variable. To query the extent to which our results generalized beyond this dependent variable, we substituted our dependent variable for two other variables that measured the content of Google search behavior in separate models: *COVID-19 aggregate vaccine relative search volume* and *COVID-19 vaccine safety relative search volume*. Those analyses are contained in Table S4.

Table S4: Robustness checks for variables that measure the content of COVID-19 vaccine searches on Google while omitting staffed intensive care beds in the U.S., January 2021 to April 2023.^a, b, c^

| **Independent Variables** | | **Aggregate Vaccine Relative Search Volume**  **Value** | ***P* value** | **Vaccine Safety Relative Search Volume**  **Value** | ***P* value** |
| --- | --- | --- | --- | --- | --- |
|  | Monthly change in Vaccine Enthusiasts | -5.084 (1.837) | .006 | 6.890 (0.583) | <.001 |
|  | Monthly change in Wait and See group | 12.510 (3.670) | .001 | -0.221 (.946) | .815 |
| ***Health care capacity, internet access, and COVID-19 variables*** | | | | | |
|  | Mean internet access (natural log) | -7.215 (2.748) | .009 | -0.592 (0.314) | .059 |
|  | Public health research facilities (natural log) | 1.369 (0.514) | .008 | 0.064 (0.058) | .270 |
|  | Access to primary care physicians (natural log) | 2.464 (.622) | <.001 | 0.214 (0.082) | .009 |
|  | Monthly change in COVID-19 cases per capita_t – 1_ (natural log) | 466.043  (24.051) | <.001 | 97.173 (4.691) | <.001 |
|  | Monthly change in COVID-19 vaccination doses per capita_t – 1_ (natural log) | 54.653 (7.230) | <.001 | 15.032 (1.647) | <.001 |
| ***Demographics*** | | | | | |
|  | Income per capita (natural log) | 12.415 (1.310) | <.001 | 1.530 (0.167) | <.001 |
|  | Change in ideology | 7.275 (3.209) | .023 | 0.924 (0.416) | .026 |
|  | Electoral pivot county | 1.087 (.560) | .052 | 0.150 (0.062) | .016 |
|  | Proportion who are non-Hispanic Black people | -14.695 (1.602) | <.001 | -2.400 (0.208) | <.001 |
|  | Proportion who are Hispanic people | -6.001 (1.271) | <.001 | -1.470 (0.172) | <.001 |
|  | Proportion who are non-Hispanic Asian American and Pacific Islander people | 13.005 (4.979) | .009 | 0.005 (0.667) | .994 |
|  | Proportion who are non-Hispanic American Indian and Alaska Native people | -15.118 (3.584) | <.001 | -5.015 (0.924) | <.001 |
|  | Intercept | -78.497 (14.628) | <.001 | -7.546 (1.881) | <.001 |
|  | *N* | 15,159 |  | 14,113 |  |
|  | *N*(Counties) | 587 |  | 587 |  |
|  | *R*^2^ (between) | 0.651 |  | 0.659 |  |
|  | *R*^2^ (within) | 0.874 |  | 0.914 |  |
|  | *R*^2^ (overall) | 0.859 |  | 0.907 |  |
|  | Wald Chi-Square | 63,236.03 | <.001 | 48,073.12 | <.001 |

^a^ Standard errors in parenthesis are clustered robust standard errors (CRSE).

^b^ Coefficients were computed by using a generalized least squares (GLS) panel regression with time-fixed effects.

^c^ Binaries for fixed effects were excluded from this table.

When we examined aggregate COVID-19 vaccine search behavior, we found that a change in Vaccine Enthusiasts was associated with reduced COVID-19 search volume (β = -5.084, Z = -2.77, *P* = .006). Additionally, a change in Wait and See was associated with increased aggregate COVID-19 vaccine search volume (β = 12.510, Z = 3.41, *P* = .001). When COVID-19 vaccine safety searches were considered, change in Vaccine Enthusiasts was associated with increased COVID-19 vaccine safety search volume (β = 6.890, Z = 11.83, *P*< .001). Interestingly, change in Wait and See was not statistically significant. Collectively, these results suggest that the results in the manuscript generalized to aggregate COVID-19 vaccine search volume, but not to COVID-19 vaccine safety searches.

## Robustness checks using varied operationalizations of the explanatory variables

In this section, we provide additional robustness checks of the analytic model using varied operationalizations of the covariates, including health care capacity, internet access, and COVID-19 variables (Table S5) and demographic characteristics (Table S6). Several alternate, explanatory variables that appeared in the Multimedia Appendix were perfectly colinear (VIF > 2) with explanatory variables in the manuscript. The variables that failed this test included hospitals (Model 2, Table S5), number of hospital personnel (Model 3, Table S5), COVID-19 fatalities (Model 4, Table S5), and both measures of the SVI (Table S6). Generally, the inclusion and subsequent exclusion of these alternate covariates did not substantially affect the central findings or the performance of the models, as the signs and statistical significance patterns of the results remained consistent.

### Health care capacity

Table S5 provides robustness analyses of health care capacity, internet access, and COVID-19 variables, including internet access rate, hospital infrastructure, hospital personnel, and the monthly change in COVID-19 fatalities. In the internet access model, we found that access to mobile broadband 4G internet may have driven the negative result, which was negative and statistically significant (β = -2.522, Z = -3.19, *P* = .001). In addition, the number of total hospitals was negatively associated with vaccine intent search on Google (β = -0.235, Z = -1.96, *P* = .050). However, hospital personnel per capita, and COVID-19 deaths per capita were statistically insignificant.

Table S5: Robustness checks to include alternative measures of health care capacity, internet access, and COVID-19 variables in the U.S., January 2021 to April 2023.^a. b. c^

| **Independent variables** | | **Internet Access**  **Value** | ***P* value** | **Hospitals**  **Value** | ***P* value** | **Hospital Personnel**  **Value** | ***P* value** | **COVID-19 Fatalities**  **Value** | ***P* value** |
| --- | --- | --- | --- | --- | --- | --- | --- | --- | --- |
|  | Monthly change in Vaccine Enthusiasts | -10.255 (1.287) | <.001 | -10.207 (1.289) | <.001 | -10.214 (1.289) | <.001 | -9.885 (1.289) | <.001 |
|  | Monthly change in Wait and See group | 9.079 (2.540) | <.001 | 9.169 (2.544) | <.001 | 9.177 (2.535) | <.001 | 9.319 (2.560) | <.001 |
| ***Health care capacity, internet access, and COVID-19 variables*** | | | | | | | | | |
|  | Mean internet access (natural log) | n/a |  | -2.406 (1.130) | .033 | -2.498 (1.107) | .024 | -2.571 (1.090) | .018 |
|  | Fixed internet access rate (natural log) | -0.526 (1.305) | .687 | n/a |  | n/a |  | n/a |  |
|  | Mobile broadband 4G coverage rate (natural log) | -2.522 (0.791) | .001 | n/a |  | n/a |  | n/a |  |
|  | Public health research facilities (natural log) | 0.379 (0.243) | .119 | 0.492 (0.236) | .037 | 0.445 (0.242) | .066 | 0.394 (0.233) | .090 |
|  | Access to primary care physicians | 0.907 (0.270) | .001 | 0.966 (0.286) | .001 | 0.876 (0.263) | .001 | 0.931 (0.259) | <.001 |
|  | Total hospitals (natural log) | n/a |  | -0.235 (0.120) | .050 | n/a |  | n/a |  |
|  | Total hospital staff per capita (natural log) | n/a |  | n/a |  | -127.522 (243.438) | .600 | n/a |  |
|  | Monthly change in COVID-19 deaths per capita_t – 1_ (natural log) | n/a |  | n/a |  | n/a |  | 1526.153 (786.727) | .052 |
|  | Monthly change in COVID-19 cases per capita_t – 1_ (natural log) | 124.167 (11.637) | <.001 | 123.903 (11.609) | <.001 | 123.990 (11.636) | <.001 | n/a |  |
|  | Monthly change in COVID-19 vaccination doses per capita_t – 1_ (natural log) | 25.289 (3.314) | <.001 | 25.308 (3.318) | <.001 | 25.326 (3.318) | <.001 | 25.556 (3.398) | <.001 |
| ***Demographics*** | | | | | | | | | |
|  | Income per capita (natural log) | 4.746 (0.559) | <.001 | 4.880 (0.563) | <.001 | 4.888 (0.570) | <.001 | 4.792 (0.566) | <.001 |
|  | Change in ideology | 4.056 (1.463) | .006 | 4.224 (1.469) | .004 | 4.223 (1.473) | .004 | 4.254 (1.466) | .004 |
|  | Electoral pivot county | 0.604 (0.273) | .027 | 0.565 (0.257) | .028 | 0.545 (0.263) | .038 | 0.610 (0.264) | .021 |
|  | Proportion who are non-Hispanic Black people | -4.696 (0.783) | <.001 | -4.722 (0.787) | <.001 | -4.886 (0.796) | <.001 | -4.967 (0.777) | <.001 |
|  | Proportion who are Hispanic people | -1.097 (0.572) | .055 | -0.837 (0.609) | .169 | -1.167 (0.570) | .041 | -1.140 (0.575) | .047 |
|  | Proportion who are non-Hispanic Asian American and Pacific Islander people | 2.094 (2.205) | .342 | 2.314 (2.161) | .284 | 2.163 (2.171) | .319 | 2.249 (2.155) | .297 |
|  | Proportion who are non-Hispanic American Indian and Alaska Native people | -5.716 (2.398) | .017 | -5.376 (2.333) | .021 | -5.491 (2.332) | .019 | -5.093 (2.236) | .023 |
|  | Intercept | -25.511 (6.223) | <.001 | -26.999 (6.255) | <.001 | -27.258 (6.326) | <.001 | -25.630 (6.315) | <.001 |
|  | *N* | 13,166 |  | 13,166 |  | 13,166 |  | 13,166 |  |
|  | *N*(Counties) | 587 |  | 587 |  | 587 |  | 587 |  |
|  | *R*^2^ (between) | 0.314 |  | 0.315 |  | 0.310 |  | 0.316 |  |
|  | *R*^2^ (within) | 0.771 |  | 0.771 |  | 0.771 |  | 0.768 |  |
|  | *R*^2^ (overall) | 0.752 |  | 0.752 |  | 0.752 |  | 0.749 |  |
|  | Wald Chi-Squared | 20,766.14 | <.001 | 20,944.78 | <.001 | 20,888.73 | <.001 | 20,013.19 | <.001 |

^a^ Standard errors in parenthesis were clustered robust standard errors (CRSE).

^b^ Coefficients were computed by using a generalized least squares (GLS) panel regression with time-fixed effects.

^c^ Binaries for fixed effects were excluded from this table.

### Demographic characteristics

Table S6 summarizes robustness analyses where we regressed most of the demographic variables from our main models on the SVI and high-risk county measures. Both measures were perfectly colinear with hospital infrastructure, non-Hispanic Black population, household income per capita, and the monthly change in COVID-19 deaths per capita. The inclusion of either measurement of social inequality or vulnerability does not affect the central manuscript findings. However, including both measures decreased the model’s performance, especially as it related to controlling for the variance between counties. Both measures were statistically significant and negatively associated with vaccine intent search behavior (continuous measure: β = -4.426, Z = -13.48, *P*< .001; high-risk county binary measure: β = -2.209, Z = -11.21, *P*< .001). These results suggest that people living in counties with the highest need of vaccine protection were also among those least likely to make vaccine intent searches.

Table S6: Robustness checks to include measures for populations at risk during public health emergencies in the U.S., January 2021 to April 2023.^a, b, c^

| **Independent variables** | | **Continuous SVI**  **Value** | ***P* value** | **High-Risk County Binary**  **Value** | ***P* value** |
| --- | --- | --- | --- | --- | --- |
|  | Monthly change in Vaccine Enthusiasts | -10.516 (1.288) | <.001 | -10.489 (1.288) | <.001 |
|  | Monthly change in Wait and See group | 9.024 (2.556) | <.001 | 8.931 (2.578) | .001 |
| ***Health care capacity, internet access, and COVID-19 variables*** | | | | | |
|  | Mean internet access | -3.842 (1.247) | .002 | -3.618 (1.326) | .006 |
|  | Public health research facilities (natural log) | 0.783 (0.232) | .001 | 0.612 (0.245) | .012 |
|  | Access to primary care physicians (natural log) | 1.422 (0.185) | <.001 | 1.343 (0.204) | <.001 |
|  | Monthly change in COVID-19 cases per capita_t – 1_ (natural log) | 124.662 (11.714) | <.001 | 122.362 (11.760) | <.001 |
|  | Monthly change in COVID-19 vaccination doses per capita_t 1_ (natural log) | 25.136 (3.329) | <.001 | 25.412 (3.359) | <.001 |
| ***Demographics*** | | | | | |
|  | Social vulnerability index | -4.426 (0.328) | <.001 | n/a |  |
|  | High-risk county | n/a |  | -2.209 (0.197) | <.001 |
|  | Change in ideology | 6.351 (1.620) | <.001 | 6.001 (1.715) | <.001 |
|  | Electoral pivot county | 0.559 (0.301) | .063 | 0.654 (0.345) | .058 |
|  | Proportion who are Hispanic people | 2.438 (0.538) | <.001 | 1.440 (0.544) | .008 |
|  | Proportion who are non-Hispanic Asian American and Pacific Islander people | 8.072 (1.669) | <.001 | 10.206 (1.915) | <.001 |
|  | Proportion who are non-Hispanic American Indian and Alaska Native people | -1.907 (1.870) | .308 | -2.547 (1.970) | .196 |
|  | Intercept | 27.690 (0.976) | <.001 | 25.838 (1.017) | <.001 |
|  | *N* | 13,166 |  | 13,166 |  |
|  | *N* (Counties) | 587 |  | 587 |  |
|  | *R*^2^ (between) | 0.227 |  | 0.182 |  |
|  | *R*^2^ (within) | 0.771 |  | 0.771 |  |
|  | *R*^2^ (overall) | 0.747 |  | 0.744 |  |
|  | Wald Chi-Square | 21,223.73 | <.001 | 21,629.74 | <.001 |

^a^ Standard errors in parenthesis were clustered robust standard errors (CRSE).

^b^ Coefficients were computed by using a generalized least squares (GLS) panel regression with time-fixed effects.

^c^ Income per capita and the proportion who are non-Hispanic Black people are excluded from this analysis because they were highly correlated with both measures of the SVI. Binaries for fixed effects were excluded from this table.
